# Templated dewetting: designing entirely self-organized platforms for photocatalysis

**DOI:** 10.1039/c6sc02555b

**Published:** 2016-08-09

**Authors:** Marco Altomare, Nhat Truong Nguyen, Patrik Schmuki

**Affiliations:** a Department of Materials Science , Institute for Surface Science and Corrosion WW4-LKO , University of Erlangen-Nuremberg , Martensstraße 7 , D-91058 Erlangen , Germany . Email: schmuki@ww.uni-erlangen.de ; Fax: +49 9131 8527582 ; Tel: +49 9131 8527575; b Chemistry Department , Faculty of Sciences , King Abdulaziz University , 80203 Jeddah , Kingdom of Saudi Arabia

## Abstract

Formation and dispersion of metal nanoparticles on oxide surfaces in site-specific or even arrayed configuration are key in various technological processes such as catalysis, photonics, electrochemistry and for fabricating electrodes, sensors, memory devices, and magnetic, optical, and plasmonic platforms. A crucial aspect towards an efficient performance of many of these metal/metal oxide arrangements is a reliable fabrication approach. Since the early works on graphoepitaxy in the 70s, solid state dewetting of metal films on patterned surfaces has been much explored and regarded as a most effective tool to form defined arrays of ordered metal particles on a desired substrate. While templated dewetting has been studied in detail, particularly from a mechanistic perspective on lithographically patterned Si surfaces, the resulting outstanding potential of its applications on metal oxide semiconductors, such as titania, has received only limited attention. In this perspective we illustrate how dewetting and particularly templated dewetting can be used to fabricate highly efficient metal/TiO_2_ photocatalyst assemblies *e.g.* for green hydrogen evolution. A remarkable advantage is that the synthesis of such photocatalysts is completely based on self-ordering principles: anodic self-organized TiO_2_ nanotube arrays that self-align to a highest degree of hexagonal ordering are an ideal topographical substrate for a second self-ordering process, that is, templated-dewetting of sputter-deposited metal thin films. The controllable metal/semiconductor coupling delivers intriguing features and functionalities. We review concepts inherent to dewetting and particularly templated dewetting, and outline a series of effective tools that can be synergistically interlaced to reach fine control with nanoscopic precision over the resulting metal/TiO_2_ structures (in terms of *e.g.* high ordering, size distribution, site specific placement, alloy formation) to maximize their photocatalytic efficiency. These processes are easy to scale up and have a high throughput and great potential to be applied to fabricate not only (photo)catalytic materials but also a large palette of other functional nanostructured elements and devices.

## Abstract 2

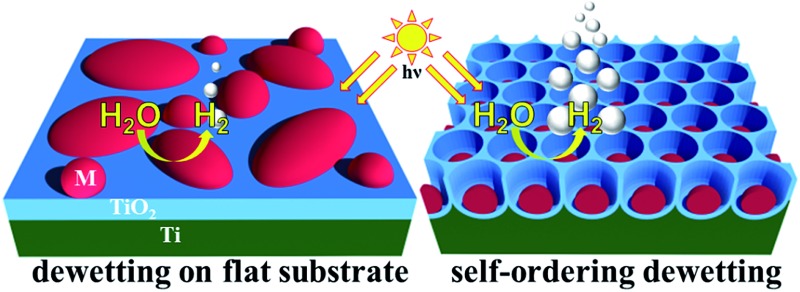
Noble metal dewetting on self-organized TiO_2_ nanotubes – nanoscopic design of photocatalysts towards green H_2_ generation.

## 1. Introduction

Defined metal (M) particles of small size scale decorating an oxide surface are of wide technological interest and find application in catalysis (chemical, electro- and photo-), photonics, plasmonics, and sensing among other areas.^1,2^

**Fig. 1:**
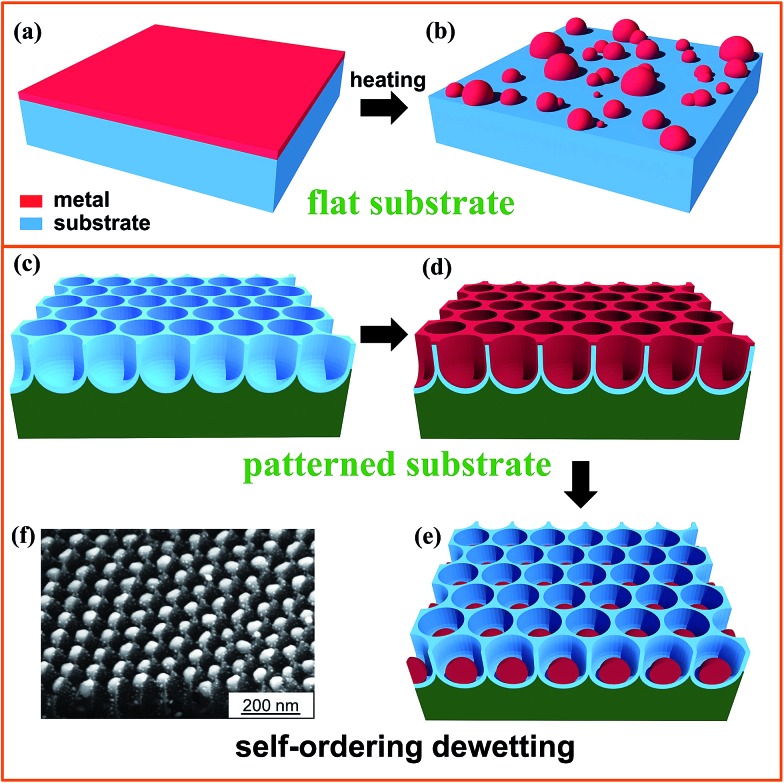
Sketch illustrating dewetting of a metal film (a, b) on a flat surface and (c–e) on a patterned surface of TiO_2_ nanotubes (*i.e.*, template-guided dewetting); (f) SEM image of arrays of single-Au-particle-per-cavity formed by dewetting a Au film on a highly regular self-organized anodic TiO_2_ nanocavity layer. Fig. (f) is reproduced with permission from ref. 16.

One way to produce such particles or ensembles is by “dewetting” a thin metal film, as illustrated in Fig. 1a and b. For virtually all metal/oxide combinations, a thin metal (some nm – to hundreds of nm) deposited by any classic method (chemical or physical vapour deposition, sputtering, evaporation, *etc.*) will, upon heating to elevated temperatures (as a rule of thumb half the metal’s melting point), break up into patches and “fingery networks”, and finally aggregate into distinct individual patches or particles (Fig. 1b).^3,4^

Except for this purely thermally driven dewetting, there are also other mechanisms such as electrotransport, where metal atom migration is caused by an applied electric field. Applied gradients that influence surface diffusion can give to dewetting a direction such as in thermotransport where the mass transport takes place along a temperature gradient across the metal film.^5^

By modification of the initial metal layer thickness, surface wettability, chemical or physical inhomogeneity of the film/substrate ensemble, and specifically by a defined pre-patterning of the substrate (Fig. 1c and d), it is possible to steer the geometry of the dewetting process into highly defined arrangements (Fig. 1e) – an example is given in Fig. 1f, where the SEM image shows a gold layer that was dewetted on a self-organized titania nanotube (NT) surface forming highly monodisperse single Au nanoparticles (NPs) of ∼60 nm in diameter in each and every titania cavity.

It is interesting to note that the same driving forces that can be beneficially explored to produce these highly defined metal particles in nanotube arrays were historically firstly reported as an undesired phenomenon. Particularly the occurrence of solid-state dewetting of thin metal films or interconnects in micro-electronic and integrated systems may lead to metal rupture or discontinuities in a metal contact and thus can result in total device failure.^5–9^

The underlying forces that separate a thin film into small islands can also lead to agglomeration of very small metal particles (for example, nm sized catalyst particles that are pre-decorated on a substrate) into coarser aggregates or patches – in catalysis coarsening of the catalyst is, of course, undesired as it causes activity degradation.^10^

In any of these examples the overall driving force for dewetting is the minimization of the free surface energy of the metal film, of the substrate and of the metal–substrate interface. Given that the thinner the metal film the higher its surface-to-volume ratio and thus its surface energy, the driving force for dewetting increases dramatically when the film thickness decreases (*i.e.*, the thinner the metal film the lower the activation energy for metal atom surface mobility). This is a key reason why dewetting can occur at temperatures that are well below the film melting point, that is, the film can dewet while remaining in the solid state.

While a range of factors (discussed in Section 3) influence initiation and growth of discontinuities even in “ideal” thin films, most metal deposition conditions lead to a meta-stable state for the as-deposited films, as they are formed under conditions for which the atomic motion is limited and thus an equilibrated lattice may not be achieved.^3,4^

Key elements for initiation of dewetting in films on ideal surfaces are defects (inhomogeneities) such as holes, edges, impurities, and grain boundaries. However, dewetting can be intentionally brought about at specific sites by using a pre-patterned substrate; that is, dewetting can be driven by a defined substrate surface topography rather than by random intrinsic inhomogeneities.

After giving some examples (below) the present perspective will give a brief overview of the general features of the dewetting process, and of key parameters and key possibilities to generate desired dewetting patterns. Emphasis will then be on metal/oxide combinations, and particularly on forms of dewetting that lead to combinations of metal/oxide structures with synergetic effects that can be used in various applications. In particular we discuss pathways to exploit dewetting phenomena for chemistry, (photo-)electrochemistry, catalysis, and some other applications. We will discuss the dewetting of (noble) metals on highly regular self-organized anodic TiO_2_ nanotubes that yields strongly enhanced photocatalytic properties of M/TiO_2_ combinations. In this context, even more complex assemblies can be fabricated introducing additional self-ordering principles, such as controlled dealloying, spinodal decomposition, site-selective functionalization and others.

## 2. Some examples

**Fig. 2:**
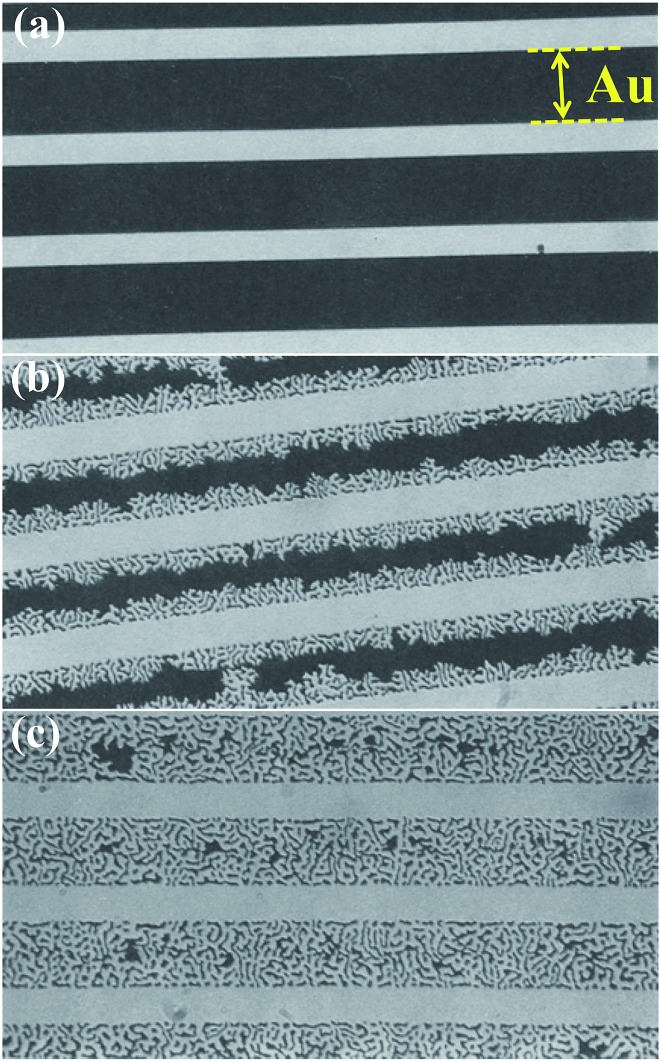
Example of dewetting of a Au line-patterned film on a fused silica substrate: (a) before annealing; (b) partially agglomerated; (c) fully agglomerated. The Au line-patterned film (dark area) is 40 to 90 nm thick. The Au lines are 25 μm in width and their spacing (bright area) is 10 μm. The Au line edges recede as shown in (b) until the line is completely transformed into islands or beads (c). For a fixed temperature the film transformation rate (expressed as area fraction transformed over time) is constant. The intensity of the laser light transmitted through the Au film was used to measure the extent of agglomeration upon annealing as a function of time. The film transformation rate depends also on film thickness and annealing temperature. Fig. (a–c) are reproduced from ref. 11 with permission of Springer.

Historically, a large effort in dewetting research addressed the agglomeration of metal films, metal silicides and metalloids on silicon and SiO_2_ (silicon-on-insulator (SOI) structures) with the goal of suppressing dewetting^5–9^ (an example is shown in Fig. 2 where the agglomeration of Au lines leads to break up of an interconnect^11^).

However, a steadily increasing number of works demonstrate that the spontaneous dewetting of metal thin films can also be exploited to a technological advantage. For example, in 2001 Liu *et al.* found that 5–10 nm-thick co-deposited layers of Au and Pd that had undergone thermal dewetting on Si wafers, agglomerated into defined metal islands that could then be used as catalytic sites for the growth of arrays of amorphous silicon nanowires.^12^ Early studies on solid-state dewetting demonstrated that the initial metal film thickness determines key structural parameters of the dewetted metal islands (*e.g.*, their size, spacing and density).^13^ Liu *et al.* adjusted the thickness of the deposited Au–Pd film to control size and distribution of the Au–Pd islands and gained in turn fine control over the morphology and coverage of the formed Si nanowires.

Meanwhile, a similar strategy was applied by Chhowalla *et al.* to the growth of vertically aligned carbon nanotubes from dewetted nickel films.^14^ They demonstrated that the structure, degree of ordering and mechanism of formation of the carbon nanotubes can be easily controlled by tuning the initial thickness of the Ni films.

The fine control over size and distribution of the dewetted films was exploited to assemble non-volatile memory devices based on arrays of dewetted Si-nanocrystal that showed tuneable stored charge density.^15^ Other work showed that dewetting of metals also represents a powerful tool for the fabrication of a large palette of micro- and nano-structured assemblies such as catalysts and electrodes,^16^ sensors,^17^ nanocrystals for magnetic elements,^18,19^ and biomimetic and plasmonic platforms.^20–22^

Another key direction of efforts was devoted to find a reliable pathway to maximize the self-ordering degree of dewetting. Among several experimental parameters that have been explored (*e.g.*, physico-chemical properties of the metal film, thermal treatment conditions, *etc.*), the topography of the substrate was identified as the most influential factor.

Some pioneering works on “templated dewetting” reported on metal films that were dewetted on pre-patterned substrates, such as grating structures,^23^ and arrays of pits and mesas^24^ in order to produce metal structures with a high degree of control over periodicity and arrangement, and to understand the underlying mechanism.^25^ For this, patterned SiO_2_/Si substrates were used due to the availability of well-established lithographic tools that allow for patterning of the substrate with nanoscale precision.

## 3. Some key concepts of solid-state dewetting

In principle dewetting involves the formation of a hole in a thin film that reaches the substrate and the subsequent recession of the film (Fig. 2) – such an initiation process can be observed for solid and liquid thin films. Solid films can be composed of, *e.g.*, polymers, metalloids or metals. Metal films can be amorphous or crystalline. Basically also single crystals on atomically flat surfaces are subjected to initiation and spreading of rupture as a consequence of self-induced spinodal (stochastic) instabilities.^4,26–32^

In practice, however, distinct (deterministic) initiation sites for film rupture are brought about by inherent defects or inhomogeneities^3^ – such as impurities, thickness variations and particularly grain boundaries in polycrystalline metal films.^11,33–37^

Once a film rupture event occurs, thin solid films deposited on a foreign substrate are generally unstable (except for cases of perfect wettability). In particular, any metal/oxide combination does have a driving force for dewetting by the reduction of the total free energy provided namely by a reduction of the interfacial area between the film and its substrate, that finally results in agglomeration of the film into three-dimensional (3D) islands. The thermodynamic driving force can be defined as:^3,4^

1 *E*_S_ = *γ*_A_ + *γ*_AB_ – *γ*_B_

where *γ*_i_ is the surface energy density of the material (with i = A (film), B (substrate)) and *γ*_AB_ the interfacial energy density.

**Fig. 3:**
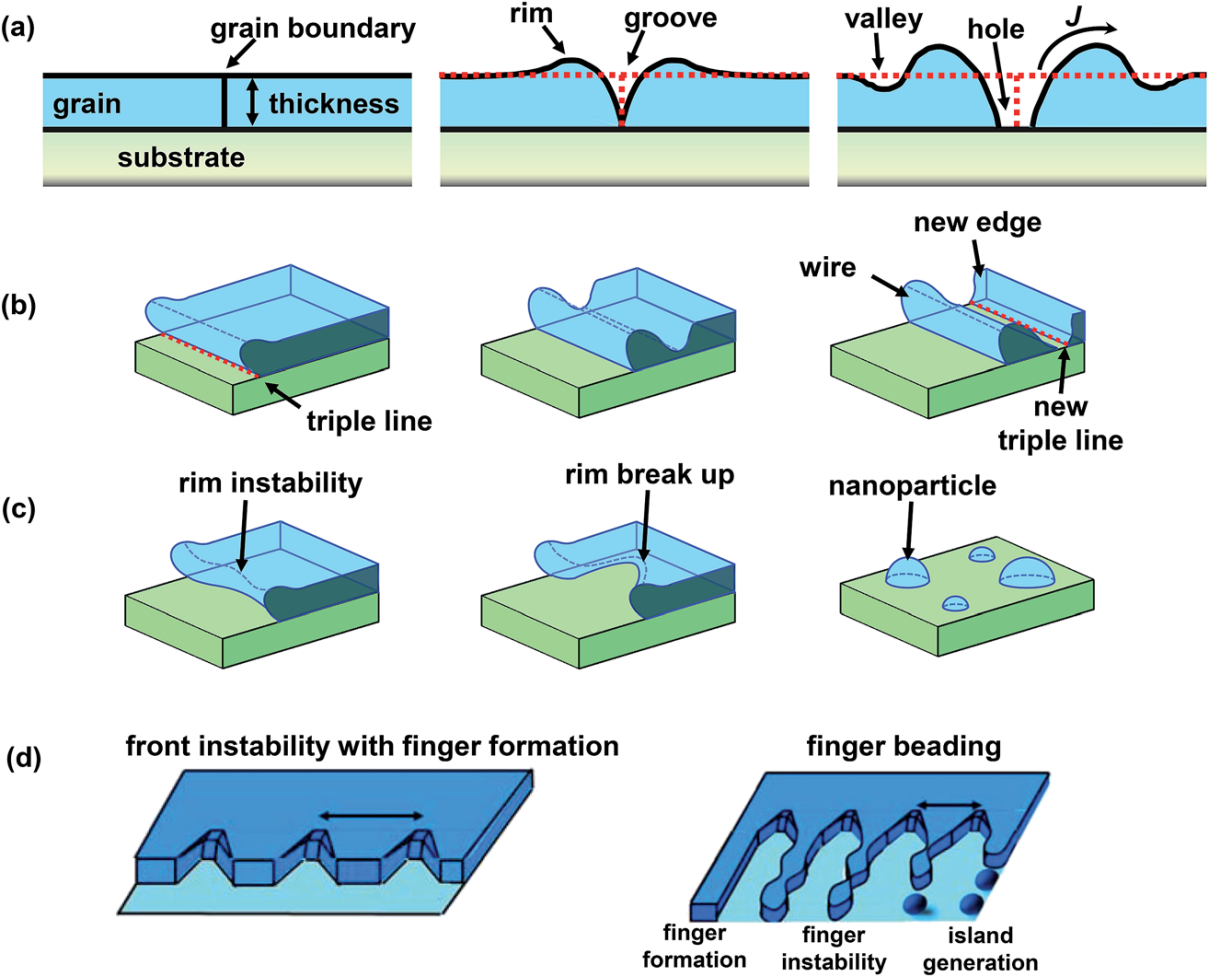
Sketch of the mechanism of solid state dewetting of a metal thin film on a smooth surface illustrating: (a) grain boundary grooving and hole formation; (b) film edge retraction and rim pinch-off; (c) rim instability and break up; (d) finger formation and beading. Fig. (d) is reprinted from ref. 4 with permission from Elsevier.

The solid state dewetting process itself proceeds from the spontaneous formation of voids or holes at specific defects (Fig. 3a) and *via* a flux (*J*) of material A leaving the dewetting zone by capillary-driven surface diffusion;^11,33,35^ *i.e.*, *J* is driven by the local surface curvature of the receding metal film. Due to mass conservation, the metal first accumulates into a rim at the dewetting front. According to its crystallographic properties the rim may be smooth or faceted and its height depends on the dewetting velocity and the kinetics of diffusion of adatoms on the surface of the film. As the rim is receding, a valley is generally formed behind the rim due to a Rayleigh instability (Fig. 3a and b). When the bottom of the valley reaches the underlying substrate, a pinch-off mechanism occurs leaving a line (wire) of material A behind (mass shedding^35,38^ – Fig. 3b). In absence of stabilizing factors, the retracting rim at a certain length becomes unstable too (Fig. 3c). As a result, large-scale ordered arrays of elongated structures (generally called “fingers”) are formed as the dewetting front recedes (Fig. 3c and d). Then, finally, the fingers break into 3D islands by a beading mechanism similar to a Rayleigh–Plateau transition,^39^ as sketched in Fig. 3d.

Regarding the initiation site, as mentioned, thin films deposited by evaporation and sputtering provide a number of relevant inhomogeneities.^40^ Most important, these films are typically polycrystalline in nature with average grain sizes of tens or hundreds of nanometres. The grain boundaries and particularly triple junctions are typical initiation sites for film rupture that trigger the formation of holes.^33^

The key parameters that generally determine the morphology of the dewetted state and the kinetics of dewetting for a given A/B couple are:

• The initial film thickness *h*. The density of formed holes scales inversely with the film thickness.^13,41^ The dewetting temperature *T*_dewet_ decreases with *h*.

• Capillary energies and film surface curvature. Capillary energies drive material retraction from the edges of the holes. The rate of material retraction scales with the difference of film surface curvature at the film edge and away from the hole (*i.e.*, where the film is virtually flat).^34^

• The treatment temperature *T* primarily affects the kinetics of the system by the adatom mobility that increases exponentially with increasing *T*. In experimental observation, metastable films dewet forming metal islands when heated up to temperatures that allow for sufficiently high surface mobility of the constituent atoms. As a result, there is a characteristic temperature for thin metal films at which dewetting can be observed, that is, *T*_dewet_ is empirically found to be between the Hüttig and Tammann temperatures that are 0.3 and 0.5 of the melting point of the metal, respectively (*T*_Hüttig_ is the temperature at which atoms at defects become mobile, and *T*_Tammann_ is the temperature at which atoms in the bulk metals start to diffuse).^42^ *T*_dewet_ is also found to decrease with decreasing metal film thickness, as a lower thermal budget (*i.e.*, lower energy barrier) is required to initiate film breakup.^3,13^ In general, the higher the metal melting temperature (*T*_m_), the higher *T*_dewet_. However, *T*_dewet_ depends also on the grain structure evolution of the metal film. Grain growth in high-purity metallic films can occur at temperatures as low as 0.2*T*_m_. In contrast, grain growth in pure diamond cubic materials occurs only when *T* ∼ 0.8*T*_m_ or higher.^3,43^ The metal surface self-diffusivity can be affected by inclusion of dopants,^5,6,44^ or by the environment (annealing atmosphere, vacuum, *etc.*).^45–48^

• The thermodynamic driving force *E*_S_. This parameter essentially depends on the couple A/B (although it may be modified by a foreign adsorption or by strain). The interface tensions *γ*_x_ are material specific.^49–52^

• Strain affects the kinetics. Films are often in a state of mechanical stress and can also experience strain effects when annealed on a substrate due to differential thermal expansion.^53^ Srolovitz *et al.* found that when holes form in a film in a state of strain, the strain in the material adjacent to the hole can partially relax.^54^ The decrease in strain energy associated with the presence of the hole makes hole formation more likely, and thus it can favour dewetting.

• The nature and number of defects. Defects in the film (pinholes and edges, dislocations, thickness variations, impurities) act as nucleation centres for spontaneous void opening.^55^ Typically the higher the kinetic energy used for a metal film deposition (and the deposition rate), the larger the density of defects in the film.^40^ Also, the thinner the film (*e.g.*, few nanometres), the larger the density of pinholes. A large density of holes accelerates the kinetic of dewetting.

• The crystallographic features. Although the concepts of capillary forces and surface curvature have in principle no proper physical meaning in the presence of singular flat facets, the crystallographic orientations of the dewetting edges and the faceting of the rim can influence the stability conditions of the dewetting front.^56–58^ These features play an important role in the morphology of the final dewetted state as well as on the dewetting kinetics. Experiments showed that, *e.g.*, edges with different in-plane orientations retract at different rates, and differently faceted rims may induce various dewetting morphologies.^59,60^

• Triple line pinning. The specific properties of the triple line, related for instance to local adsorption and/or local pinning ascribed to defects, can affect the local mobility of the dewetting front.

**Fig. 4:**
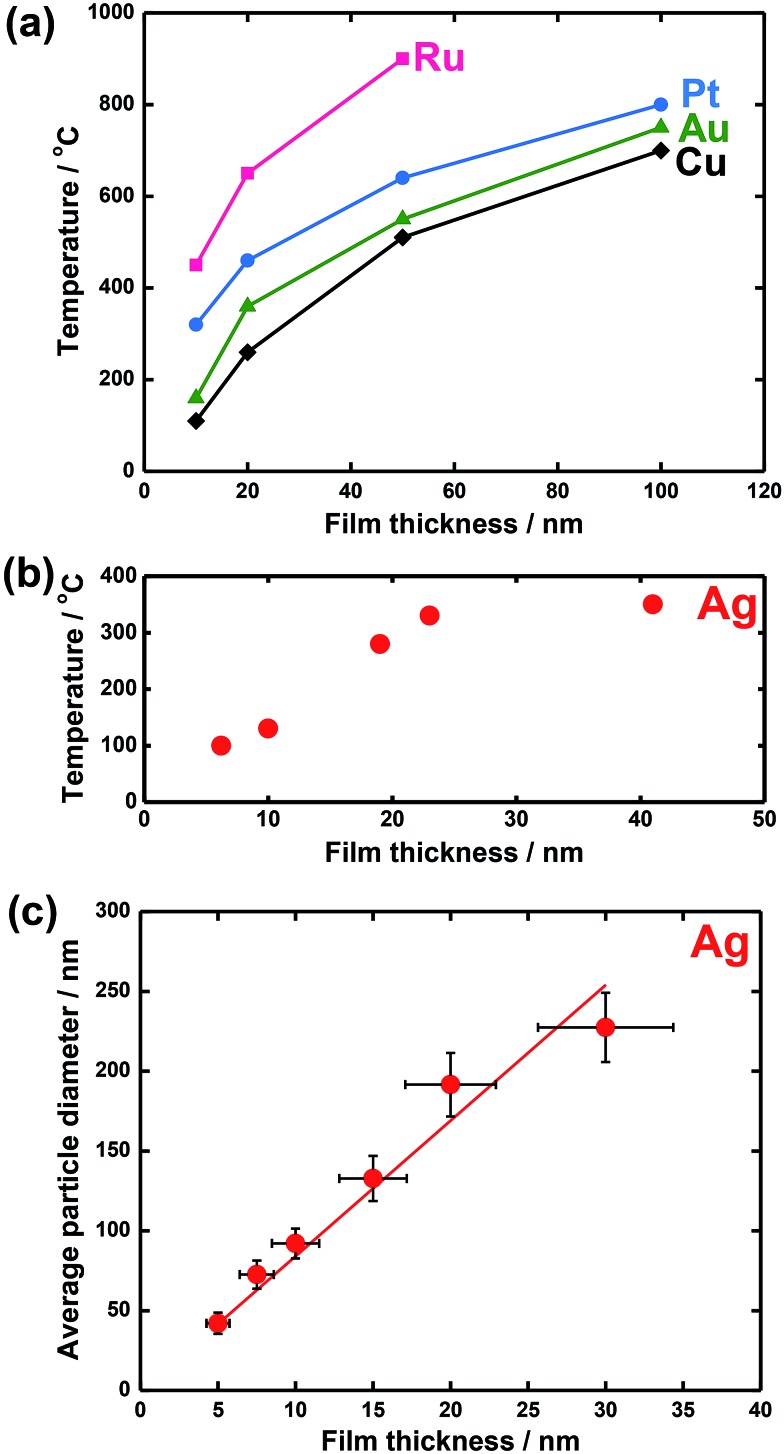
Some examples of parameter effects: *T*_dewet_ for (a) island formation in Ru, Pt, Au, and Cu films and (b) in a Ag film as a function of film thickness; (c) average particle size for fully dewetted Ag films as a function of film thickness. Fig. (a) is reprinted with permission from ref. 61. Copyright 2005, American Vacuum Society; Fig. (b) is reproduced from ref. 62 with permission of Springer; Fig. (c) is reproduced from ref. 63 with permission of IOP Publishing.

However, from an experimental point of view it is generally difficult to discriminate these parameters. Some examples of parameter effects are shown in Fig. 4.^61–63^

In summary, an ideal polycrystalline film on a smooth substrate undergoes full dewetting, forming islands of metal, the shape, size, spacing and density of which are relatively homogeneous throughout the surface of the initially continuous film.

However, common thin films show intrinsic defects from which dewetting preferentially initiates. Hence, extensive material agglomeration starts not only at grain boundaries but also at pre-existing holes and film edges. One consequence is that the kinetics of the process is no longer governed only by the hole incubation time.^3,11,13,64^ A second consequence, which is more relevant in the frame of this perspective, is that the intrinsic defects in metal films can cause a certain loss of self-ordering degree of dewetting so that the metal may agglomerate into islands of irregular shape and size and with random distribution. Nevertheless defined defects (pre-patterned substrates) can be used beneficially to achieve a highly controlled dewetting process as discussed in the next section.

## 4. Templated dewetting

**Fig. 5:**
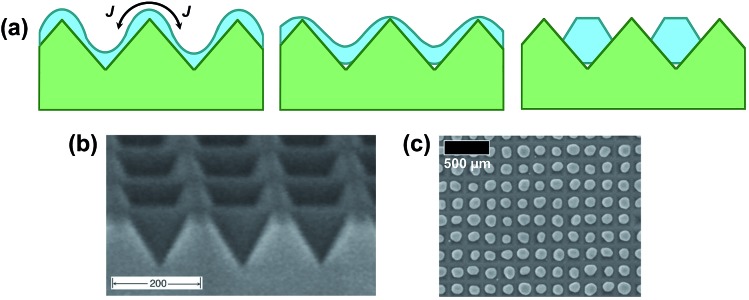
Mechanism and example of templated dewetting: (a) schematic cross-sectional view of metal film dewetting on a patterned surface. *J* is the curvature-driven atomic flux on the surface; SEM micrograph of (b) a square-array of inverted pyramids in (100) silicon and (c) ordered arrays of one metal particle per pit (with no extraneous particles) formed by self-ordering dewetting of a 21 nm-thick Au film on an array of inverted pyramids with a 175 nm period. Fig. (b, c) are reprinted from ref. 24 with the permission of AIP Publishing.

The idea of templated dewetting is to impose an initial periodic perturbation in the film curvature to control breakup and the subsequent metal morphological evolution. An important factor that determines if dewetting can be controlled by the substrate to take place in an ordered manner is the topography features compared to film thickness, *e.g.*, in a ripple structure, as in Fig. 5a, the thickness of the deposited layer needs to be in the range of the ridge height, and the wavelength of the ripples and distances covered by atomic motion need to be of similar magnitude.^23,24,65–68^

For two-dimensional (2D) patterned surfaces (Fig. 5) the control over the NP size can be achieved if the introduced topography has an artificial curvature modulation with a shorter length scale than the natural instability on a flat surface – this results in a decay into smaller particles.^24^ In this case the curvature-induced diffusion mechanism dominates over other agglomeration dynamics (*e.g.*, driven by capillary instability or grain growth) as long as the ridges can act as diffusion barriers that trap the metal into the valleys – under these conditions agglomeration is limited and the surface coverage stagnates.

Interestingly, it was observed that dewetting on corrugated surfaces occurs at lower temperatures compared to flat substrates. It was thus proposed that the ripple patterned topography provides a gradient of chemical potential in the direction normal to the direction of the ripples (with minima in the valleys and maxima at the peaks) that triggers curvature induced diffusion (*J* in Fig. 5a).^67,69^ Fine control of the NP size can thus be achieved by film thickness and substrate topography.^42,68,70–77^

Another key to ordered dewetting is the match between the geometry of the trenches (*e.g.*, the angle at their bottom) and the crystallographic features of the dewetted metal crystals (as shown in Fig. 5a).^23,25^ Evidently, if too-thick metal films (*i.e.*, larger metal amounts) are deposited that fill the trenches completely, one may essentially lose the corrugation effect during the early stage of dewetting, as a virtually even surface is created.^67^

### 4.1 Some examples of templated dewetting

A pioneering study on template-guided dewetting of metal using diperiodic substrates is the work of Giermann and Thompson.^24^ They explored the formation of ordered metal nanoparticles by dewetting Au films on square-arrays of pyramidal pits formed on SiO_2_/Si surfaces (Fig. 5b). Different topographical geometries were used that had various spatial period and pit-to-mesa width ratios. It was found that for specific ranges of relative Au film thickness and topographic dimension, dewetting resulted in arrays of nanometre-scale Au particles embedded in each pit, with highly-uniform periodic spacing, of nearly monodisperse size and controlled crystallographic orientation – *i.e.*, Au dewetted in a completely complementary manner with respect to the substrate topography.

Another remarkable work is that of Kushida *et al.* In this study, polycrystalline Pt films were evaporated and then annealed onto oxidized Si surfaces that had been previously lithographically patterned with sawtoothed grating structures.^23,66^ Owing to the specific angle at the bottom of the trenches, it was found that for a certain thickness of the metal film and by a suitable annealing treatment the initially conformal Pt film decomposed, forming within each groove one-dimensional (1D) Pt crystals that had a preferential crystallographic orientation – the Pt crystals grew with the (111) plane parallel to the faces of the grooves.

Such a template-guided grain growth was discussed as driven not only by the metal film but also by the formation of stabilized Pt crystals within the trenches (Fig. 5a). In contrast, a similar annealing of Pt carried out on smooth SiO_2_ surfaces led to irregular Pt particles having no specific preferential crystallographic orientation.

### 4.2 Other interesting aspects

Choi *et al.* found that both the initial metal thickness and temperature of thermal treatment provide control over dewetting.^70^ However, not only does the former lead to better size control of the dewetted NPs, but also annealing at too-high temperature (used in principle to cause crystal coarsening) may lead to a loss of metal by evaporation.

Kojima and Kato developed a technique to form periodically arranged metal NPs by electron-beam-induced dewetting.^63^ The main advantages of the technique are that one can select the region where to generate the particles, and that such a region has sharp boundaries (*i.e.*, between dewetted and non-dewetted areas).

Dewetting can also be triggered particularly using nanosecond pulsed lasers.^76,77^ This method allows also for dewetting of high melting point metals and avoids substrate deterioration (*e.g.*, by thermal oxidation). Ruffino and Grimaldi provide an overview of different heat sources (annealing, ion or electron beam, laser irradiation) that can be used to cause dewetting.

Oh *et al.* found that on a topographic substrate (arrays of pits) one particle per pit is obtained when the spacing between the pits is similar to the average distance between the NPs dewetted on a smooth surface (from a metal film of similar thickness).^71^

Yang *et al.* explored templated-dewetting of co-sputtered metal films.^42^ The use of different metals in controlled amounts leads to simultaneous alloying and dewetting, and forms *e.g.* ordered alloyed Au–Ag particles with fined composition. They also showed that further control over size and spacing of the metal particles is obtained by using the effect of gravity and by an iterative deposition-dewetting approach.

Overviews of various other metal/substrate combinations that have been investigated are also available in the literature.^4,78^ It is however remarkable that while templated dewetting has been studied from a mechanistic perspective in detail, the resulting potential for applications is only considered in comparably few works, such as the fabrication of Si nanowire arrays,^70^ magnetic nanocrystals,^71^ optical platforms (exhibiting, *e.g.*, enhanced plasmonic properties, surface localised plasmon resonance (SLPR) and surface-enhanced Raman scattering (SERS)),^42,73^ and electrocatalysts.^74^

In the following section we will deal with using dewetting of (noble) metals on self-organized TiO_2_ nanotubes, where such a combination is entirely application driven by the functional features of noble metal decorated TiO_2_, namely for photocatalysis.

## 5. TiO_2_ nanotube surfaces for self-ordering dewetting

We have outlined above how the use of periodic surfaces can lead to controllable dewetting to form defined arrays of metal NPs on a regular substrate. This approach is and has been studied using patterned SiO_2_/Si substrates due to the availability of well-established lithographic tools that allow for patterning the substrate (with large scale uniformity) to virtually any geometry and length scale.^24,68^

**Fig. 6:**
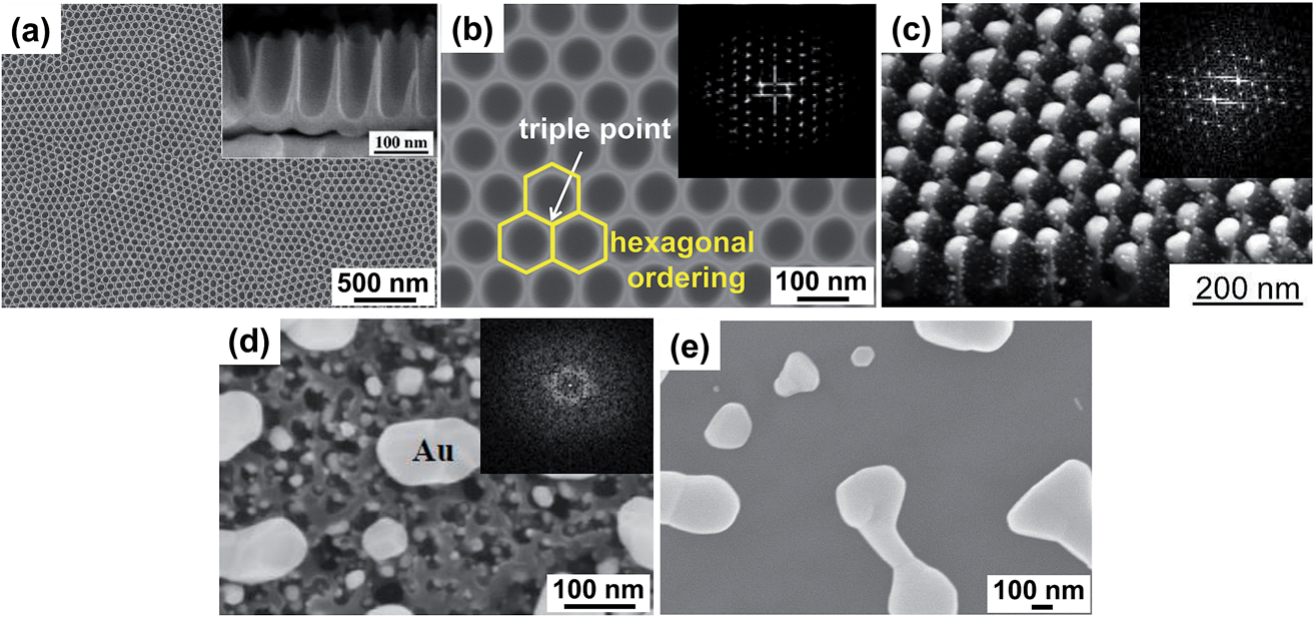
(a) SEM images of a highly ordered TiO_2_ nanocavity array (individual cavity length *ca.* 200 nm, top width *ca.* 80 nm, wall thickness *ca.* 15 nm) produced on a Ti substrate by self-organizing anodization in hot 3 M HF/H_3_PO_4_ electrolyte (top and side view); (b) high magnification SEM image of TiO_2_ cavities showing the ideally hexagonal packing (inset: FFT conversion of the SEM image); (c) example of self-ordering templated dewetting of Au film (20 nm-thick) on a highly ordered TiO_2_ nanocavity array leading to total filling with exactly one metal nanoparticle per TiO_2_ cavity (inset: FFT conversion of a top-view SEM image of array of highly-ordered Au NPs embedded in TiO_2_ nanocavities). (d, e) Example of non-ideal conditions for dewetting of a 20 nm-thick Au film: SEM image of metal dewetting on (d) an insufficiently ordered nanotube surface (TiO_2_ NTs formed by anodization of Ti in an aqueous mixture of NaH_2_PO_4_ and HF at 20 V for 2 h; inset: FFT conversion of a top-view SEM image of TiO_2_ NTs prior to Au sputtering–dewetting); and on a TiO_2_ compact flat anodic oxide surface (formed by anodization of Ti in 0.5 M aqueous H_2_SO_4_ at 20 V 30 min). Fig. (a–e) are reproduced with permission from ref. 16.

The use of self-organizing patterns for templated dewetting only recently received wider attention, this because emerging self-organizing electrochemical techniques applied to namely Al, Ti and Ta provided reliable tools to fabricate patterned metal oxide substrates with a sufficiently high degree of ordering.^79,80^ TiO_2_ nanotube arrays that can reach the highest degree of self-ordering were reported by Yoo *et al.* in 2013 (Fig. 6a and b).^16^ These nanotubes are almost ideally hexagonally ordered and are of a suitable short aspect ratio to provide a periodic surface for ideal ordered dewetting (Fig. 6c).

Below, we briefly introduce the fabrication process and geometry of these anodic TiO_2_ nanotubes (that we refer to also as TiO_2_ nanocavity arrays). Then we introduce noble metal dewetting on these arrays and show the highly beneficial use of these metal/oxide assemblies in photocatalysis.

### 5.1 Highly-ordered anodic TiO_2_ nanotube arrays

A direct, scalable and versatile approach to form self-ordered titania structures (but also of other metal oxides^81^) is self-organizing electrochemical anodization of a Ti metal substrate.^82–84^ Vertically aligned regular TiO_2_ nanostructures with defined tubular geometry can be formed and the self-ordering degree, morphology, and physicochemical properties of the TiO_2_ nanotubes can be adjusted by choosing an adequate set of electrochemical parameters.^85,86^ Several literature reviews are available that discuss in detail the formation and properties of these anodic nanotubes^82–84,87^ – therefore we will keep this part very brief.

The key to a high degree of self-ordering, as shown in Fig. 6a and b, is the use of electrochemical conditions that during anodization lead to high rate of oxide growth combined with high rate of oxide dissolution.^88,89^ This can be achieved by anodizing Ti metal in hot H_3_PO_4_/HF electrolytes. The tube growth conditions can be adjusted to form a short tube length that resembles a nanocavity. The resulting arrays of TiO_2_ nanotubes can be formed over large surfaces (some cm^2^) and present a virtually ideal hexagonal ordering (Fig. 6a and b).^16^

Onto these TiO_2_ nanocavity surfaces one can deposit metal films conformally, *e.g.*, by sputtering, and then trigger dewetting by thermal treatment (Fig. 1c–f and 6c). For the subsequent dewetting to be controllable, the regular tube geometry together with an optimized cavity spacing and height relative to the deposited metal thickness is essential.

Tube arrays of a poor degree of self-ordering lead to metal dewetting in a highly imperfect fashion (Fig. 6d). Fig. 6e shows for comparison also the dewetting result on a flat substrate. FFT (Fast Fourier Transform) conversion of the SEM images (insets in Fig. 6b–d) is the most direct method to characterize the regularity of the TiO_2_ cavities and offers a clear comparison between different self-organized nanotube structures.

### 5.2 Metal/TiO_2_ structures for photocatalysis

Why are metal particles (particularly noble metal) on TiO_2_ so important? Because they provide a highly synergistic platform for photocatalysis.^90–95^

In general, a photocatalytic process is based on the interaction of light with a semiconductor immersed in a suitable reaction environment. Photons of sufficient energy promote electrons from the valence band (VB) to the conduction band (CB); this creates electron–hole pairs (e^–^–h^+^). Then, holes and electrons can be separated, reach the surface of the semiconductor and be captured by reactants in the environment. Holes can be used to oxidize suitable species while electrons cause reduction reactions.^91^

Photocatalysis has gained much attention in recent decades, mainly in view of using solar energy for degradation of pollutants, and generation of energy carriers such as hydrogen gas^96^ and hydrocarbons.^97^ The most relevant TiO_2_-mediated photocatalytic reactions are:

H_2_ generation2
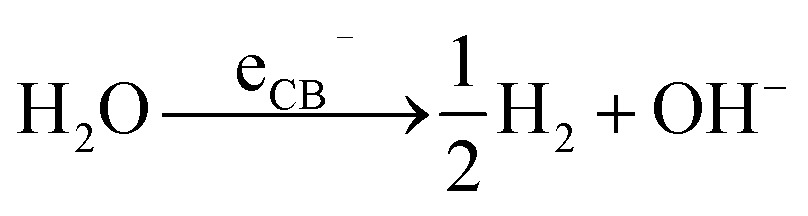



O_2_ generation3
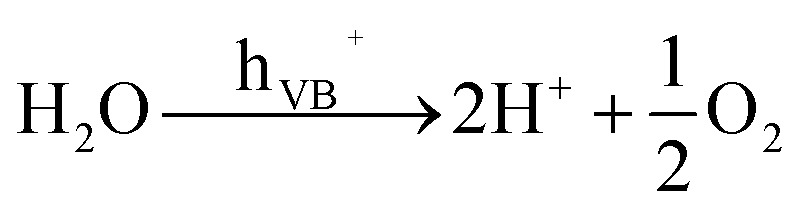



HO˙ radical generation4
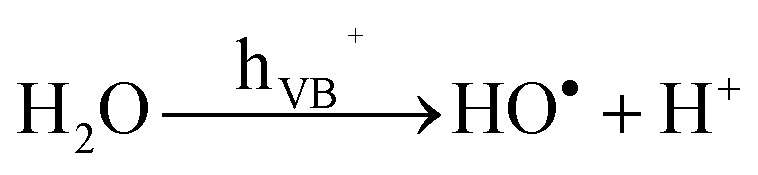



Hydrocarbons mineralization5
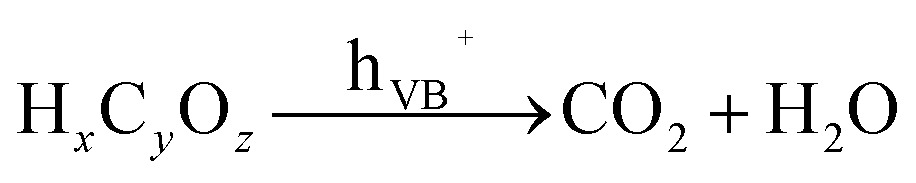



In particular, the photocatalytic generation of H_2_ from renewable sources (*e.g.*, water or water–alcohol mixtures) is of high interest.^98^ For this, the most-used semiconductor is TiO_2_. The reason is that titania, particularly in its anatase polymorph, offers a number of beneficial features such as an adequate alignment of its CB energy relative to the electrochemical potential of H_2_ generation from water, an outstanding (photo-)chemical stability, low cost and large availability.^99^

In titania, under open circuit conditions, after photoexcitation and charge carrier separation, electrons and holes can generate cathodic and anodic sites at different parts of the TiO_2_ surface. The cathodic sites are directly responsible for reduction reactions. In the case of the water splitting reaction, CB electrons can reduce water at these cathodic sites and form H_2_ as outlined in reaction (2). On the other hand, reactions (3)–(5) take place at the anodic sites and are mediated by VB holes.

However, the transfer of charge carriers from VB and CB to the redox species in the environment is kinetically hindered.^90–93,95,100^ Thereby, charge carrier can recombine (heat is released) resulting in loss of photo efficiency. Organics, such as alcohols (*e.g.*, methanol, ethanol), are commonly added to the reaction environment since they can be easily oxidized by VB holes (as so-called sacrificial agents reacting eventually towards CO_2_).^101–103^ This reduces the charge recombination, and therefore improves the lifetime of CB electrons and thus leads to an enhanced H_2_ evolution.^91,104^

More importantly, to reach reasonable H_2_ generation rates the surface of TiO_2_ needs to be modified by depositing small amounts of suitable charge transfer cocatalysts. Typical cocatalysts for TiO_2_ are noble metals such as Au, Pt and Pd.^93,105^ The noble metal particle at the TiO_2_ surface forms a Schottky-type junction that increases dramatically the overall photocatalytic efficiency by trapping the conduction band electrons (this limits charge recombination), and by mediating their transfer to the environment, *e.g.*, H_2_O. Additionally, some noble metals such as Pt aid the recombination reaction of H^0^ atoms to H_2_ gas (cocatalysis).^99^

Most commonly, TiO_2_ photocatalysts are based on nanoparticle slurries or compacted nanoparticle layers, and are decorated by noble metal particles using colloidal solutions or by (photo-)reduction from metal ion solutions.^106,107^ Owing to the nature of these methods, the metal NPs are decorated at the TiO_2_ surface in a fairly inhomogeneous way, *i.e.*, a site-unspecific manner. In the following sections we outline efforts during the past three years to use noble metal dewetting principles on TiO_2_ nanotube arrays such as in Fig. 1d–f specifically to design novel photocatalytic platforms.

### 5.3 Factors to optimize the photocatalytic efficiencies

The TiO_2_ surface geometry in the form of highly regular TiO_2_ nanocavities is not only key for controlled dewetting of the cocatalytic noble metal into NPs of desired size, density and placement, but can also offer an adjustable and in this case ideal “reaction vessel geometry” for UV-based photocatalysis and for reactions (2)–(5) as discussed above.

**Fig. 7:**
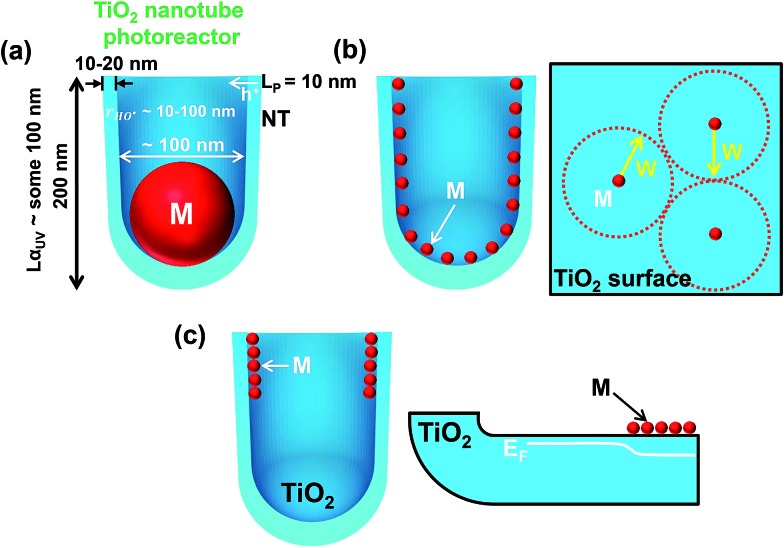
Sketch of tube geometry parameters and possible metal/TiO_2_ nanotube photocatalyst geometries obtained under different sputtering–dewetting conditions (M = metal): (a) one-cocatalyst-particle per cavity. *L*_α_UV__ = average UV light penetration depth in TiO_2_; *L*_P_ = length of holes (h^+^) solid-state diffusion in TiO_2_; *r*_HO˙_ = typical diffusion lengths of generated HO˙ radicals in the liquid phase; (b) homogeneous metal decoration. *W* = width of the space charge layer induced in TiO_2_ by the Schottky junction (*i.e.*, metal/TiO_2_ junction) and determined using the Mott–Schottky equation as discussed in ref. 111 and 112; and (c) top-only metal decoration and effect on the electronic properties of TiO_2_ nanocavities. *E*_F_ = Fermi level of the metal oxide semiconductor.

As outlined in Fig. 7a, the depth of the cavity (*i.e.*, the length of the tube sidewalls) is in the order of the UV light penetration depth in titania. The thickness of the TiO_2_ tube walls is ∼10–20 nm, which is thus comparable to the solid-state diffusion length of holes, and allows for their efficient transfer to the environment. The virtual volume of the reaction phase in each cavity (with an inner diameter of ∼80–100 nm) matches well the typical diffusion lengths of generated HO˙ radicals in the liquid phase.^16^

On these nanotubes the sputtering–dewetting conditions can be adjusted (see below) to obtain also other M/TiO_2_ configurations, as illustrated in Fig. 7b and c.

Fig. 7b illustrates the results of dewetting very thin noble metal films (with a nominal thickness of 1–2 nm) on the surface of the nanocavities. This leads to a low NP loading on TiO_2_, which allows for a maximized free TiO_2_ surface and light absorption by the semiconductor (the shadowing effect ascribed to the cocatalyst decoration is negligible).

Dewetting of thicker conformal metal films may on the one hand limit the free TiO_2_ surface (necessary for hole transfer to the environment) and also the photon flux to the semiconductor (owing to the shadowing effect), but on the other hand may provide the required density of M/TiO_2_ junctions at the TiO_2_ surface for efficient electron trapping and transfer to the environment.

From a metal/semiconductor junction viewpoint, the principle is that the particle spacing (*i.e.*, decoration density) needs to be adjusted to an optimum value so that the width of space charge layer (*W*) induced by neighbouring M NPs overlap with each other (see the model in Fig. 7b).^108,109^ *W* is defined as:6
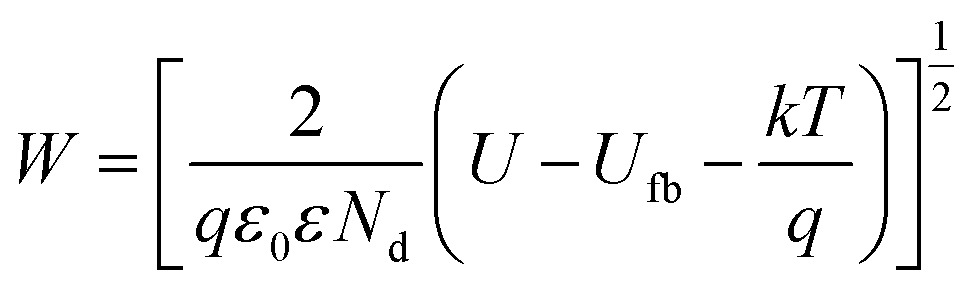
where *ε* denotes the dielectric constant, *ε*_0_ the vacuum permittivity, *q* the charge of the electron, *N*_d_ the donor concentration (for an n-type semiconductor), *U* the applied potential, *U*_fb_ the flat-band potential, *k* the Boltzmann constant, and *T* the absolute temperature.

For annealed TiO_2_ nanotubes, and other anodic anatase layers, values of *ε* ∼ 20–80 and *N*_d_ ∼ 5 × 10^18^ to 5 × 10^19^ cm^–3^ are typically reported. *U*_s_ is the difference between the flat-band potential of TiO_2_ and the work function of Au, *i.e.*, *U*_s_ ∼ 0.7 V.^110^ With typical values of TiO_2_, *W* is in the order of ∼15–30 nm.^111,112^

From a practical point of view, the most effective M cocatalyst decoration, that can be obtained by controlled metal sputtering–dewetting (and other self-ordering tools illustrated below), must be then attributed to a minimized noble metal amount that provides at the same time an optimum of key geometrical and thus electronic features of the semiconductor, namely, the ratio between free TiO_2_ surface and the area coated with cocatalyst NPs (both necessary for hole and electron transfer, respectively), light harvesting *vs.* shadowing effect, and the density of induced Schottky junctions.^111–114^

The metal decoration can also be adjusted to deposit (by shallow-angle sputtering) the cocatalyst NPs only at the mouth of the tubes (illustrated in Section 5.5). As sketched in Fig. 7c, the site-selective decoration can induce a gradient of the semiconductor Fermi level (*E*_F_) in the tube walls along the length of each TiO_2_ cavity.

The absorption depth (into TiO_2_ nanotubes) of light with an energy in the band-gap region of anatase is a few μm,^115,116^ and anatase tubes provide an electron diffusion length in the range of several 10 μm.^117^ As in a classic photocatalytic configuration the tube mouths (tube/environment interface) are directly irradiated, the site-specific noble metal deposition at the upper part of the nanocavities can be the most efficient geometry. An electron harvesting (tube bottom)/charge-transfer activity (tube top) combination can be established to significantly contribute to an overall H_2_ evolution enhancement: the transfer of electrons that are generated in the tube bottom towards the metal/TiO_2_ coupled zone (photocatalytically active zone) may be facilitated by the fact that a beneficial electronic junction is formed (*i.e.*, a gradient of *E*_F_ along the TiO_2_ tube walls).^118^ Nevertheless, this configuration provides also direct light irradiation of the TiO_2_/M/environment interface, and charge carriers formed in its close proximity can thus be effectively transferred to reactants.^119–122^

### 5.4 Orderly-dewetted Au nanoparticles/TiO_2_ nanocavities

A combination of particular interest for photocatalytic H_2_ generation is Au/TiO_2_. Au, compared to Pd and Pt, has a lower melting point (∼1064, 1555 and 1768 °C, respectively). Temperatures as low as 400–450 °C provide the required activation energy for Au surface diffusion so that Au crystals can grow through mass transport^123^ and Au films dewet into equilibrium structures.

An additional advantage is that Au films do not react with oxygen, that is, they can be dewetted in air.^6,46^ This is important for two aspects: (i) the dewetted particles maintain their metallic state (Au^0^), which is essential to form effective Au/TiO_2_ Schottky junctions;^124^ (ii) a thermal treatment at 450 °C (in air) not only leads to Au dewetting but also converts the as-formed amorphous anodic nanocavities into crystalline TiO_2_ composed of mainly anatase (*i.e.*, the most photo-active titania polymorph^125,126^).

A simple way to control the size/density of the dewetted Au NPs and their self-ordering degree is to adjust the deposited Au-layer thickness *t*_Au_ relative to the topographical features of the nanocavities (in general, the amount of deposited noble metal is expressed as “nominal thickness”). Additionally, the sputtering configuration can be calibrated with respect to the geometry of the cavity.

**Fig. 8:**
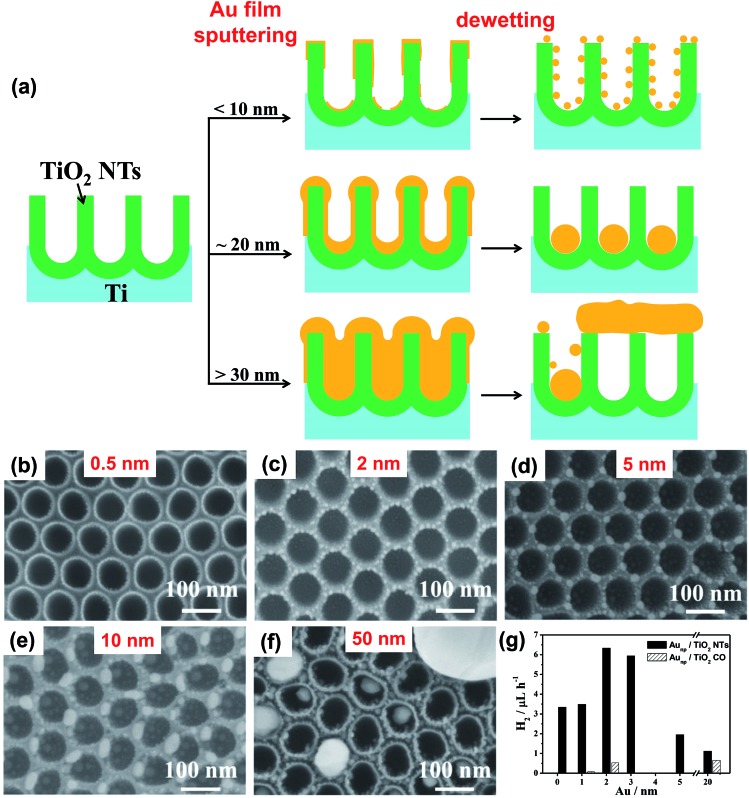
(a) Sketch of various sputtering–dewetting conditions and resulting metal/TiO_2_ nanocavity structures depending on the loading of sputter-coated metal; (b–f) SEM images of TiO_2_ nanocavity arrays decorated with Au particles by sputtering–dewetting different metal amounts, in terms of nominal initial thickness of the film – (b) 0.5, (c) 2, (d) 5, (e) 10, (f) 50 nm; (g) photocatalytic H_2_ generation data in terms of *r*_H_2__ (9-h long runs) of various Au/TiO_2_ structures. Black columns are for Au/TiO_2_ nanocavity arrays and striped columns are for anodic TiO_2_ compact oxide (TiO_2_ CO) decorated with Au. Fig. (b–g) are reprinted from ref. 127 with permission from Elsevier.

As shown in Fig. 8a for relatively thin sputtered films (<10 nm), the metal can be deposited mainly at the rims and bottoms of the cavities with a sputtering direction normal to the periodic TiO_2_ surface (when avoiding rotation or tilting of the substrate). In this case the as-deposited Au coating is not continuous as almost no metal is found along the inner sidewalls of the cavities.

Fig. 8b–f shows the different geometries that can be obtained from 0.5–50 nm-thick Au films.^16,127^ Films with *t*_Au_ ∼ 0.5–1 nm already partially dewet in the as-deposited state, *i.e.*, without annealing, forming discontinuous films with nm-sized cracks (*T*_dewet_ is possibly < room temperature). However, a clear change of morphology is observed after the thermal treatment. Films with *t*_Au_ ∼ 0.5 dewet forming Au NPs that are round in shape and with average diameter of 2 nm (the NP size/distribution is homogeneous throughout the TiO_2_ surface – Fig. 8b).

Thicker Au films (with *t*_Au_ up to ∼10 nm) show a clear interaction with the periodic titania substrate. Au layers of 2–3 nm split into circular arrangements of ∼5 nm-sized NPs that decorate the rim of the nanocavities (Fig. 8c). Dewetted 5 nm-thick Au films (Fig. 8d) form ∼5–6 nm NPs that are arranged in a hexagonal network (mirroring the hexagonal-packing of the TiO_2_ cavities). Each Au NP is located atop the cavity triple-point, *i.e.*, where the sidewalls are shared by three adjacent cavities (Fig. 6b). This strong metal–substrate interaction occurs because not only do the edge of the rims provide a positive excess of chemical potential,^24^ but also because *t*_Au_ (5 nm) is comparable in size to the width of the cavity sidewalls (10 nm).^68^

A different result is observed at the bottom of the TiO_2_ cavities. These locations provide a smooth surface with low curvature and no sharp edges. As a consequence Au layers with *t*_Au_ < 5 nm are more likely to dewet as on a flat ideal surface, and the NP size and density depends only on the Au initial thickness (as reported for smooth substrates^3,68^).

When the Au layers are thicker, one observes an inversion of this trend. For *t*_Au_ ≥ 10 nm (Fig. 8a and e), the Au films dewetted at the top of the rims with a loss of self-ordering degree. The hexagonal arrangement is lost, the NP spacing is inhomogeneous and their size distribution becomes broad. The situation is opposite in the nanocavities. 10 nm-thick Au films split in 3–4 particles of uniform size (10–12 nm) that are confined closed to each other at the very bottom of the cavity.

A remarkable result is found for 20 nm-thick Au films which provide ideal conditions for maximized self-ordering, leading to arrays of ∼50 nm-sized single Au cocatalytic NPs per each photocatalytic TiO_2_ cavity (Fig. 1f and 6c).^16^ The fabrication process is highly reliable and the arrays are filled with almost 100% success rate over large surfaces (some cm^2^).

In line with the concept outlined in Section 4, this result is ascribed to the synergistic interplay between the geometry of both the TiO_2_ cavities and Au film.^24,63^ In spite of the orthogonal sputtering configuration, as-deposited 20 nm-thick Au films coat the periodic substrate virtually in a conformal way (Fig. 8a) – the film is continuous along the TiO_2_ surface (with only small fluctuations in its thickness) which is key to controllable dewetting. Initially, the sidewalls of the cavities act as pre-defined locations for the rupture of the metal film, and the TiO_2_ rims are exposed to the ambient.^65,67^ Then, Au dewetting proceeds independently in each cavity and the negative excess of chemical potential causes complete Au retraction from the sidewalls towards the very bottom of the cavity.^24^

The effect of the highly-ordered TiO_2_ surface is remarkable and can be assessed comparing such a result to compact anodic TiO_2_ films loaded with a similar amount of Au and dewetted accordingly (Fig. 6e). Notably, the Au NPs embedded in the periodic TiO_2_ substrate are not only much smaller than those on a flat surface but are also monodisperse in size (sharp size distribution), and their spacing (particle-to-particle distance) is one order of magnitude smaller than on a flat substrate.

This means that this approach can provide Au NPs at the TiO_2_ surface with a fully tuneable decoration density (typically much higher than obtained on smooth TiO_2_). The fine control over the Au/TiO_2_ structures is a key prerequisite for their use not only as an efficient photocatalyst (see below) but potentially also as functional electrodes, high-density memory devices, plasmonic platforms and SERS-based sensors.

Au films thicker than >20 nm agglomerate in a random fashion and with large size distribution (from few nm to few μm – as shown in Fig. 8a and f). These results are, in terms of particle size/spacing distribution, similar to those observed for Au dewetted on flat surfaces. In line with these findings,^24^ the loss of self-organization is ascribed to the excessive metal initial thickness relative to the topography features of the substrate.^67^

The photocatalytic efficiency of the Au/TiO_2_ systems was explored in terms of H_2_ generation from water–ethanol mixtures under monochromatic UV light irradiation (325 nm).^16,127^

The highest hydrogen efficiency (in terms of H_2_ evolution rate *r*_H_2__) is found for 2 nm-thick Au film dewetted on the TiO_2_ nanocavities (Fig. 8g). These arrays lead to a *r*_H_2__ of ∼6.3 μL h^–1^ that is more than 10 times higher than that of Au/TiO_2_ structures formed on a flat anodic oxide, and *ca.* 2 times higher than that of a similar sample that was not subjected to the dewetting step^120^ – the latter result confirms the contribution of dewetting to the photocatalytic enhancement.

Worth noting, for dewetted Au on compact oxide the H_2_ generation increases with increasing the Au loading (Fig. 8g – striped columns). Conversely, a remarkably lower amount of Au is required on the TiO_2_ tube arrays for maximizing the photoactivity. Interestingly, Au films which are either thinner or thicker than 2 nm lead (on the tubes) to a dramatic reduction of the H_2_ evolution rate, in line with discussion in Section 5.3.

Moreover, repeated photocatalytic runs and photocurrent measurements under external bias-free conditions showed the Au/TiO_2_ systems to be highly stable, and neither significant poisoning nor deterioration of the cocatalyst/catalyst took place with their prolonged use.^16^

### 5.5 Adding dealloying to form nanoporous Au/TiO_2_ nanocavities

**Fig. 9:**
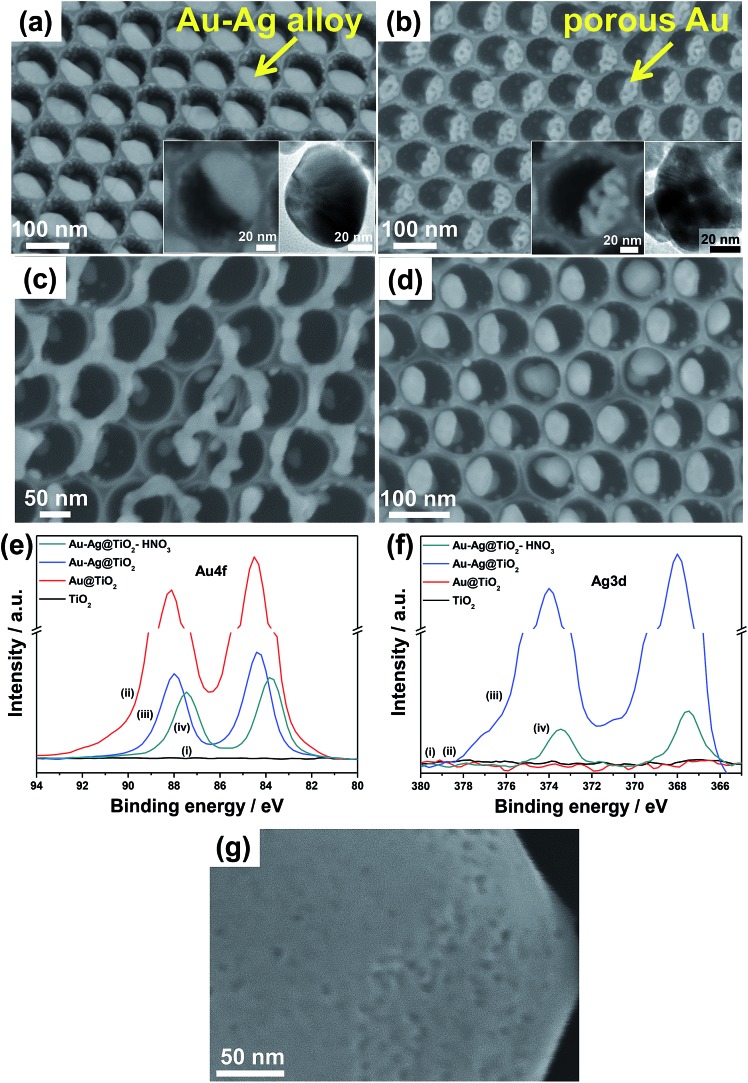
(a–d) SEM images of (a) Au–Ag/ and (b–d) various Au/TiO_2_ nanocavity arrays structures formed by (b, d) dewetting–dealloying and only (c) dealloying. (a) Arrays of single Au–Ag alloyed NPs per cavity after thermal dewetting in Ar (400 °C) of sequentially sputter-coated Au and Ag films and (b) arrays of single porous Au NPs after dealloying in HNO_3_ (insets in (a) and (b): high magnification SEM and TEM images of (a) Au–Ag alloyed NP and (b) porous Au NP after dealloying). (c, d) Non ideal dewetting–dealloying conditions: TiO_2_ nanocavities decorated by dealloying of Au 10 nm to Ag 20 nm film (c) without dewetting process and (d) after dewetting in air. (e, f) XPS spectra of the (e) Au and (f) Ag regions of: (i) as-formed TiO_2_ NTs, (ii) Au (1 nm)/TiO_2_ NTs, (iii) dewetted Au (1 nm)–Ag (2 nm)/TiO_2_ NTs and (iv) dewetted–dealloyed Au (1 nm)–Ag (2 nm)/TiO_2_ NTs. (g) SEM image of TiO_2_ compact layer decorated by dewetting and dealloying (in HNO_3_) a Au 10 nm–Ag 20 nm film. Fig. (a–c and e–g) are reproduced with permission from ref. 120.

An approach to maximize the cocatalyst specific area is dealloying, *i.e.*, to maximize the Au/environment interface. This can of course be used in the context of dewetted particles on TiO_2_ tubes too by suitable dewetting (and then dealloying) a cocatalyst/sacrificial metal combination on the TiO_2_ nanocavities (Fig. 9a and b).

Dealloying is widely explored as a nanoscale processing tool to fabricate ultra-high surface area metals for various applications (catalysis, sensing, optical applications). As such, it consists of the selective dissolution of the more (electro-)chemically active element of a single-phase alloy. Typically it leads to the formation of a nanoporous continuous metal sponge that can be almost entirely composed of the more noble element. As a consequence, the activity of the metal per loaded mass can be dramatically improved.^128–131^

Key parameters are the composition and structure of the initial metal alloy. A simple sequential sputtering approach of two (or more) metals combined with thermal dewetting is an efficient approach to form an alloy at the surface of the TiO_2_ substrate. The sputtered metals can be selected so that one is less noble than the other(s). The thermal treatment then not only forms the metal alloy precursor of desired composition necessary for the subsequent dealloying step, but also splits the metal alloy into fine NPs of controllable size and distribution.^42^

A suitable alloy for this purpose is Au–Ag. The relatively low melting point of these two metals (Ag melts at ∼962 °C) is a crucial advantage since a single optimized thermal treatment at ∼400 °C leads simultaneously to Au/Ag alloying and dewetting. Moreover, Ag is less noble than Au and can be selectively dissolved. Additionally, Au and Ag form alloys in any composition, *i.e.*, with no miscibility gap. These are key prerequisites in the dealloying step to achieve controllable porosification of the metal cocatalyst.^132,133^

In the case of pure Au, a 20 nm-thick sputtered film is found to dewet into arrays of single-Au-NP-per-cavity (Fig. 1f and 6c). As proposed by Giermann *et al.*,^25^ one of the conditions for maximized ordering during dewetting is that the capacity of each cavity of the periodic substrate matches the volume of metal deposited at the surface of (within) the cavity.

Remarkably, when an adequate metal loading is deposited over the TiO_2_ surface (a 10 nm-thick Au film followed by deposition of an additional 20 nm-thick Ag film), the deposited double-metal layer (having an overall nominal thickness of 30 nm) is found to dewet accordingly (in Ar, 400 °C), and split with ∼100% success rate into a single 50–60 nm-sized alloyed Au–Ag NP in each cavity (Fig. 9a).

However, for this combination of metals on TiO_2_ (compared to the case of pure Au/TiO_2_) the thermal treatment must be optimized. A most efficient solution is a sequential annealing approach. Firstly, the pristine nanocavity layer is annealed in air (450 °C) – the presence of oxygen in the annealing atmosphere leads to oxide crystallization into anatase TiO_2_ with minor content of rutile and low density of oxygen vacancies.^134–136^ Then, to effectively form an alloy that can be orderly dewetted–dealloyed, a thermal treatment in an inert atmosphere (Ar) is necessary.

Both dewetting and alloying occur through a mass transport mechanism and thus a certain surface mobility of both Au and Ag atoms is needed (this is granted by the inert atmosphere). In contrast, when annealing in air Ag/Au films on a regular TiO_2_ surface, Au agglomerates into particles (segregates) while Ag is left behind in the form of irregular strands/patches. This negatively affects the result of dealloying (no porosification) and can limit the photocatalytic performance of the metal/oxide systems – we illustrate below how the porosification of Au impacts the photocatalytic enhancement.

On the other hand, Au–Ag NPs that are alloyed–dewetted by argon-annealing into the TiO_2_ cavities can be successful dealloyed by an adequate etchant (*e.g.*, concentrated HNO_3_). This leads to the highly-defined nanoporous Au/TiO_2_ assemblies as shown in Fig. 9b.^132,133,137^

The importance of adequate dewetting conditions is remarkable since no porosification is expected to take place for Au–Ag films that are not properly alloyed. Au–Ag films deposited on TiO_2_ surfaces were subjected to dealloying either without any preliminary alloying–dewetting step or after air-annealing. In the first case, the result of etching is metal patches of irregular shape that are randomly distributed over the oxide surface. This structure is formed since no dewetting took place but only Ag dissolution (Fig. 9c). In the second case, single Au NPs per cavity are formed (note however that the success rate is lower compared to argon-dewetting). Here, Au underwent ordered dewetting (in line with our results using pure Au films) and the Ag that was left behind was then dissolved by the etchant (Fig. 9d). Most importantly, neither the first nor the second Au structure shows porosification, this confirming the importance of using a proper sequence of alloying–dewetting–dealloying.

A comparison of the insets in Fig. 9a and b shows that each single alloyed Au–Ag deposit turns into a porous particle. The pores are few nm in diameter and the average size of each particle is somewhat retained, *i.e.*, ∼50–60 nm. A key for this is that Au and Ag elements are homogeneously alloyed and orderly dewetted, as confirmed by the XPS data in Fig. 9e and f. The shift of Au and Ag XPS peaks after dewetting and dealloying confirms that both these steps lead to significant change of the chemical surroundings in the metal NPs. The Au XPS peaks of the nanoporous Au/TiO_2_ nanotube arrays show a binding energy of 83.9 eV that is similar to that reported in the literature for similar systems, *i.e.*, dealloyed porous Au.^129^

Differently, the same process carried out on a flat oxide surface leads to irregular metal patches that can be as large as several hundreds of nm (Fig. 9g). For these large patches the dealloying step leads only to some surface pores and thus the increase of metal specific surface area is almost negligible.

In practice, this alloying–dewetting–dealloying approach has great potential to fabricate nanoporous metal or alloy/TiO_2_ structures, the composition and geometry of which can be tuned by a simple bulk processes, *e.g.*, by adjusting metal loading, relative amount and deposition sequence, sputtering configuration, temperature of dealloying and its duration.^120^

**Fig. 10:**
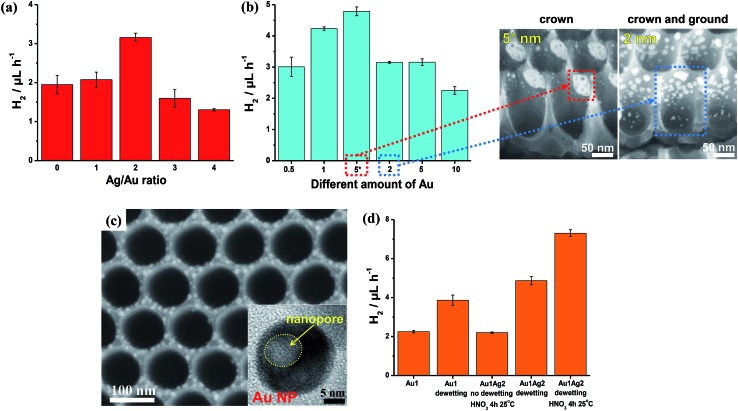
(a, b and d) Photocatalytic H_2_ evolution rate (*r*_H_2__, 5 h-long runs) measured for (a) different Ag/Au ratios (all samples were prepared by sputter-coating 5 nm-thick Au films and different amounts of Ag, followed by dewetting and a 2 h-long dealloying step at 15 °C), (b) different amounts of Au (nm) with constant Ag/Au ratio of 2 : 1 (all samples were dewetted and then subjected to a 2 h-long dealloying step at 15 °C – SEM images are relative to the samples labelled in (b) as “5*” and “2”), and (d) different Au/TiO_2_ photocatalysts prepared by depositing a 1 nm-thick layer of Au (the plot highlights that an optimized combination of dewetting–dealloying can lead to a ∼4 times increase of the H_2_ generation rate); (c) SEM and TEM (inset) images of TiO_2_ NTs decorated with porous Au–Ag NPs (by dewetting–dealloying of 1 nm-thick Au and 2 nm-thick Ag films). Fig. (a–d) are reproduced with permission from ref. 120.

Various Au/TiO_2_ assemblies were explored in view of their photocatalytic H_2_ evolution ability from water–ethanol mixtures under 325 nm UV light irradiation (in Fig. 10a and b). The results show that the key parameters for efficient H_2_ generation (*i.e.*, maximized *r*_H_2__) are the metal (Ag and Ag) loading, their relative amounts and deposition sequence.

A 1 nm-thick Au film (along with a 2 nm-thick Ag film) leads, through dewetting–dealloying, to porous Au/TiO_2_ showing a high photocatalytic performance (*r*_H_2__) owing to an optimized catalyst structure in terms of the (adequate) surface density of metal/oxide junctions and the (minimized) oxide shading effect. However, a remarkable contribution to the photocatalytic enhancement is provided by the dealloying step. Au porosification takes place even on particularly small metal particles (∼5–10 nm in size – Fig. 10c). The H_2_ generation rate of a dewetted–dealloyed sample is almost doubled (*r*_H_2__ ∼ 7.5 μL h^–1^ – Fig. 10d) compared to Au/TiO_2_ layers formed from pure Au films under otherwise identical conditions (*r*_H_2__ ∼ 4.0 μL h^–1^).^127^

Moreover, not only the metal loading but also its placement on the tubes can be adjusted by an adequate sputtering configuration. Metal layers that are deposited by a classic sputtering geometry (sputtering direction normal to the tube arrays) and then alloyed–dewetted–dealloyed form Au porous NPs either in a mixed or full crown or ground position. Whether the Au NP placement is at ground or crown position depends on the nominal metal loading (sketched in Fig. 8a), and a clearly lower *r*_H_2__ is obtained for mixed ground/crown position compared to the only crown position (Fig. 10b), which is well in line with the concepts outlined in Section 5.3.

However, the metal films can also be deposited site-specifically. For this, the tube layer substrates can be placed parallel to the direction of sputtering (shallow angle sputtering), in order to deposit the metal (Au/Ag) film only on the crown position (*i.e.*, the very top of the tubes) – then dewetting and dealloying steps follow that are carried out in otherwise identical conditions, and form dewetted–dealloyed porous Au NPs exclusively at the crown position (SEM images in Fig. 10b).

A side-effect of sputtering at a shallow angle is that the amount of Au that is actually deposited on the tubes is less than when sputtered in a normal configuration. For a nominally 5 nm-thick Au film sputtered at a shallow angle, a loading of ∼0.10 mg cm^–2^ was measured. For comparison, 1 and 2 nm-thick Au films deposited by normal angle sputtering lead to loadings of Au NPs of ∼0.07 and 0.13 mg cm^–2^ (mixed crown/ground position). The photocatalytic data in Fig. 10b clearly illustrate that the sample with crown only decoration and fabricated by shallow angle sputtering (labelled as “5*”) delivers the largest amount of H_2_. These results demonstrate the importance of a proper “positioning” of a catalytic particle if one targets the use of a minimal Au amount for achieving a maximum photocatalytic H_2_ generation performance.

### 5.6 Pt/TiO_2_ nanocavities – optimizing dewetting and oxide crystallization

The beneficial effect of Au in the photocatalytic H_2_ evolution is mainly ascribed to its ability to capture electrons from the CB of TiO_2_ and mediate their transfer to the reaction phase. For this reaction Pt is an even more efficient cocatalyst since it not only acts (as Au) as an “electron sink” but also can promote the recombination of H^0^ surface species to H_2_.^138^ This is the reason why Pt is frequently found to be more active than other noble metals (under comparable deposition conditions).^105^

Pt has a higher melting point (1768 °C) compared to Au (1064 °C), meaning that higher temperatures are required to reach sufficient surface diffusion for Pt and dewetting.^3^

Dewetting of Pt on TiO_2_ surfaces can be observed at temperatures > 500 °C;^119,121,139^ in this case the thermal treatment needs to be carried out under inert conditions (or in a reductive gas, as also reported in the literature^17,140^). If the treatment is carried out in oxygen-containing atmospheres Pt can dewet partially due to the possible reaction of Pt metal with oxygen that leads to the formation of surface platinum oxide^139^ – this may limit surface diffusion and hinder defined dewetting.^45^

**Fig. 11:**
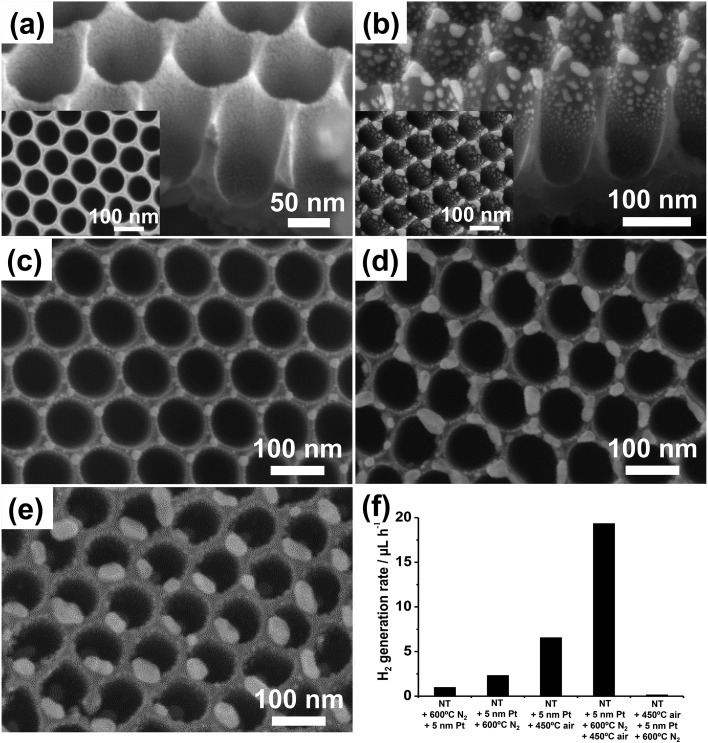
(a–e) SEM images of TiO_2_ nanotube arrays: (a) sputter-coated with a 5 nm-thick Pt film and (b) after thermal dewetting in N_2_ (600 °C); (c–e) sputter-coated with different Pt amounts ((c) 2, (d) 7, and (e) 10 nm) and then dewetted in N_2_. Insets in (a) and (b): top view SEM images. (f) Photocatalytic H_2_ generation rate (*r*_H_2__) of various Pt/TiO_2_ nanocavity arrays decorated by sputtering–dewetting of different Pt loadings. Fig. (a–f) are reprinted (adapted) with permission from ref. 139. Copyright 2016 American Chemical Society.

In pure nitrogen Pt can be dewetted (Fig. 11a–e). Fig. 11a shows the typical result of sputter-coating the TiO_2_ nanocavity substrate with a 5 nm-thick Pt film. In line with the results using Au (Fig. 8), the as-sputtered Pt film is found to coat preferentially the top of the sidewalls, and its thickness gradually decreases towards the bottom of the rims (as clear from the contrast in the SEM image). When these films are dewetted (in N_2_ at 600 °C), globular Pt NPs are formed that decorate the sidewalls and top of the tubes.

In line with the theory of dewetting, that is that particle size and spacing scale with the initial metal film thickness,^3^ Pt films of 2, 5, 7 and 10 nm in thickness form by dewetting NPs with average size of ∼5–20, 5–30, 10–40 and 15–50 nm, respectively (Fig. 11b–e). The smaller NPs are typically round in shape, show narrow size distribution, and are ordered at the tube tops in a hexagonal arrangement (Fig. 11c). Thicker metal films split into irregular Pt islands that are several tens of nm large (Fig. 11e).

Nevertheless, the photocatalytic efficiency (*r*_H_2__) of these structures is significantly lower than expected. This is clear from the photocatalytic data in Fig. 11f where the H_2_ generation rate of this sample is compared with that of a tube layer that was coated by an identical Pt film (5 nm) and then subjected to annealing in air at 450 °C (no dewetting). The latter structure leads to a 3 times higher H_2_ evolution rate. Please note also that tube layers firstly annealed in N_2_ at 600 °C and then decorated with Pt (5 nm) lead to a negligible H_2_ evolution.

The reason is the different crystallographic features of TiO_2_ NTs annealed under various conditions. Particularly, annealing treatments were found to greatly affect the degree of crystallinity of tubes and the relative amount of formed anatase to rutile with respect to the total amount of crystalline TiO_2_ (the XRD data and refinement method are discussed in ref. 139). Specifically, it was found that annealing in N_2_ forms an oxide with high degree of crystallinity (∼30 wt%) but relatively low anatase content (∼23%), and it also generates oxygen vacancies in the oxide (and a consequent photoactivity decay due to charge carrier trapping/recombination in the semiconductor^134,141^).

The solution to this problem is a multiple-step annealing firstly in N_2_ at 600 °C (dewetting) and then in air at 450 °C that leads to both high anatase relative content (∼30%) and degree of crystallization (∼29 wt%). Additionally, the XRD patterns of the structures subjected to multiple annealing (shown in ref. 139) show the characteristic reflections of Pt that can be ascribed to Pt grain growth during dewetting.

This example illustrates typical considerations when designing a dewetting experiment for functional use (photocatalysis). Here the two concepts, namely, the optimized Pt dewetting and oxide crystallization can be beneficially combined. The result is defined Pt NP-decorated TiO_2_ tube arrays where the oxide shows both a high degree of crystallization and a high relative content of anatase phase, which are necessary for photocatalytic enhancement. Also remarkable is the comparison of these photocatalytic data with the results on Au dewetting (outlined above): by using a similar sputtering–dewetting strategy, Au-modified TiO_2_ tube layers lead to a maximized photocatalytic activity of ∼6–7 μL_H_2__ h^–1^,^16,120,127^ while the Pt/TiO_2_ structures lead to a ∼3 times higher H_2_ evolution rate, *i.e.*, ∼20 μL h^–1^.

The air-crystallization step was further explored exposing dewetted layers to air annealing at various *T* (350–550 °C range), confirming that annealing at 450 °C is, in the view of photocatalytic applications, the most optimized condition. Refined XRD data show for these samples that: (i) air annealing at 350 °C does not form anatase TiO_2_ but only rutile (∼26 wt%); (ii) air annealing at 550 °C forms anatase and leads to a high degree of crystallinity of the oxide (the total content of crystalline TiO_2_ is 64.5 wt%), but it causes also the formation of a large amount of rutile (∼52 wt%).^142^ The formation at 550 °C of relatively large amounts of rutile can be due to the thermal oxidation of the Ti metal substrate. In line with previous works, this occurs firstly by rutile formation at the Ti/TiO_2_ interface and then it proceeds (with higher annealing temperatures and/or longer thermal treatments) up the tube walls and toward the tube tops.^143–146^

**Fig. 12:**
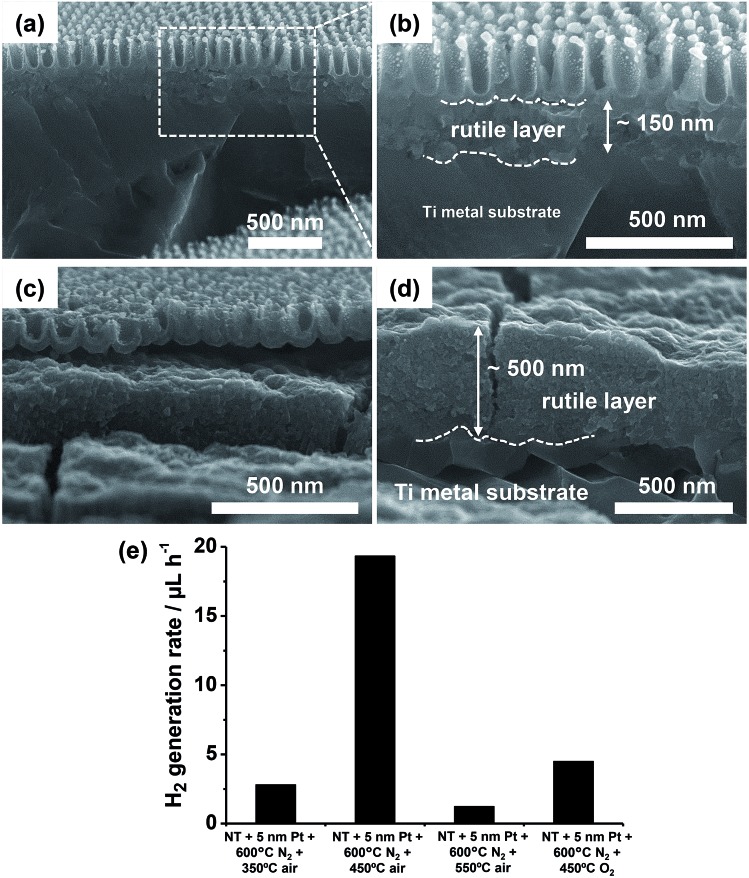
(a–d) Cross sectional SEM images showing the rutile layer formed underneath the Pt/TiO_2_ nanocavity arrays by air-annealing (after Pt dewetting) at (a, b) 450 °C and (c, d) 550 °C. (e) Photocatalytic H_2_ generation rate (*r*_H_2__) of Pt/TiO_2_ nanocavity arrays showing the effect of temperature of the post thermal treatment and use of an O_2_-containing atmosphere. Fig. (a–e) were reprinted (adapted) with permission from ref. 139. Copyright 2016 American Chemical Society.

The SEM cross-sectional images in Fig. 12a–d further confirm that rutile forms (from the Ti metal substrate) as a layer of some hundreds of nm underneath the anodic tube layer. The air treatment at 450 °C leads to a ∼150 nm thick rutile film (Fig. 12b), which is ∼3 times thinner than that formed at 550 °C (*i.e.*, ∼500 nm – Fig. 12d). Therefore, the absence of anatase phase in the layers treated at 350 °C and the predominant content of rutile in the oxides crystallized at 550 °C are the most plausible reasons for their low H_2_ generation yield.

The thermal treatment in O_2_-containing atmospheres affects not only the crystallinity of the oxide but also the oxidation state of Pt,^147^ and therefore the overall photocatalytic efficiency.^124,148^ In fact, Pt/TiO_2_ samples that were annealed after dewetting in air at too high *T* (>450 °C) or in pure O_2_ at 450 °C lead to a poor H_2_ evolution efficiency (Fig. 12e).

**Fig. 13:**
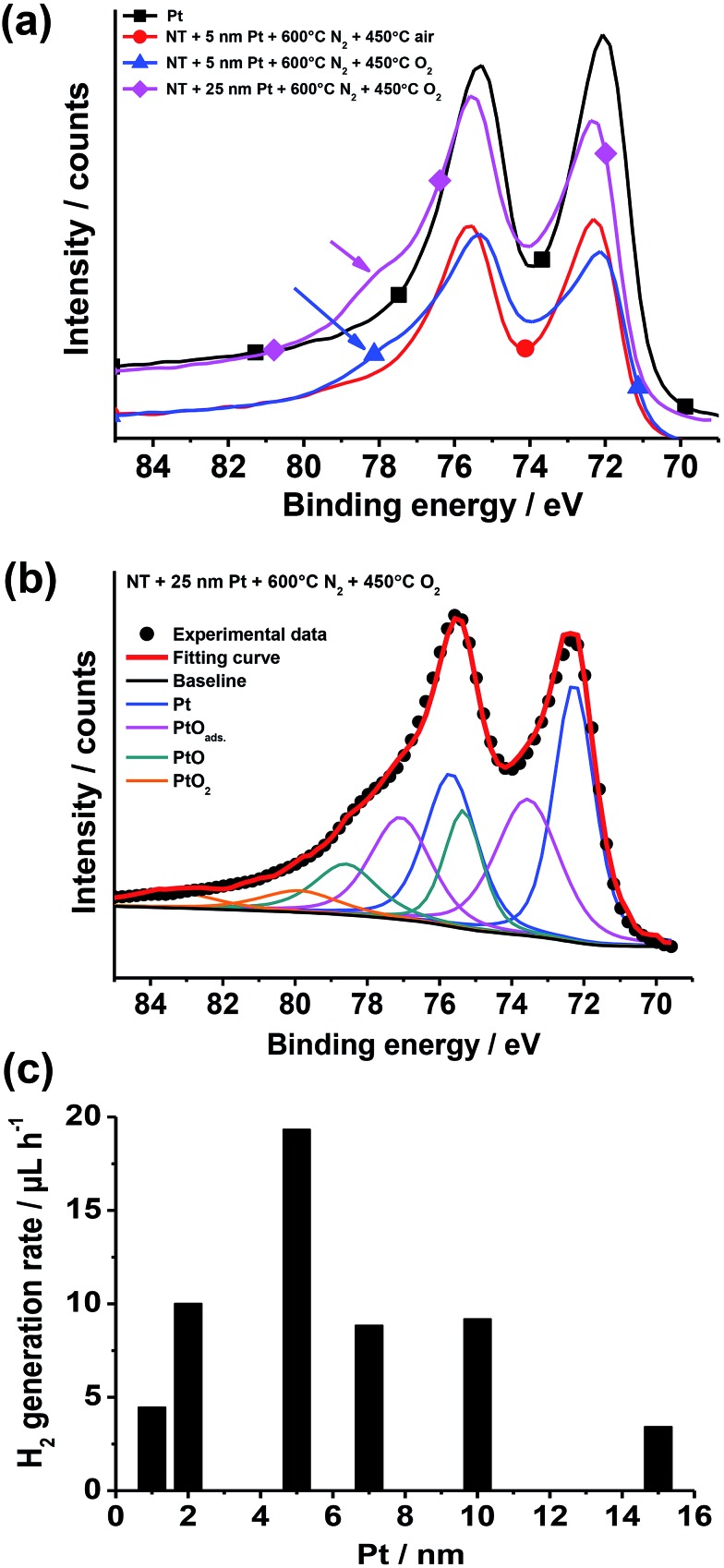
(a, b) XPS data: (a) high-resolution Pt 4f XPS spectra of Pt (reference) and of TiO_2_ nanotube layers decorated with sputter-coated Pt films (5 and 25 nm-thick) and exposed to different thermal treatments in N_2_, air and O_2_ (the arrows indicate the shoulder ascribed to Pt(ii) and Pt(iv) oxides); (b) Pt 4f high-resolution XPS spectrum (experimental data) of a TiO_2_ nanotube sample sputter-coated with a 25 nm-thick Pt film and then treated at 600 °C in N_2_ and 450 °C in O_2_ (the plot shows also the fitting curve and deconvoluted doublets accounting for Pt^0^, PtO_ads_, Pt(ii), and Pt(iv) oxides). (c) Photocatalytic H_2_ generation rate (*r*_H_2__) of Pt/TiO_2_ nanotube layers formed by sputtering–dewetting of various Pt loadings. Fig. (a–c) were reprinted (adapted) with permission from ref. 139. Copyright 2016 American Chemical Society.

XPS data (Fig. 13a and b) show that after air annealing, the noble metal at the oxide surface is present as metallic Pt (*i.e.*, Pt^0^).^148,149^ On the contrary, for Pt/TiO_2_ structures annealed in pure O_2_ a broad shoulder (at ∼76–80 eV) appears that can be attributed to the formation of PtO (PtII) and PtO_2_ (PtIV).^148^ The formation of platinum oxide is clearer when exposing tube layers coated by a relatively thick (25 nm) Pt film to O_2_ annealing for 5 h. An even more pronounced shoulder can be seen that is in line with larger amount of formed Pt oxides – fitting of this data reveals a good match with the Pt 4f reference signals of PtII and PtIV oxides.^150,151^

Thus, the low H_2_ generation efficiency of Pt/TiO_2_ structures annealed in pure O_2_ can be ascribed to the formation of Pt oxides that can limit the ability of the cocatalyst in electron trapping and transfer.^124,150–152^ Moreover, the formation of Pt oxide can explain why Pt films were found not to dewet by annealing in air or oxygen, that is, the formation of surface oxide can reduce Pt surface diffusion.^45^

The effect of the amount of Pt cocatalyst on the H_2_ evolution efficiency was also explored. The photocatalytic results, as observed in the case of sputter-dewetted Au/TiO_2_ layers,^16,120,127^ show a clear enhancement of the H_2_ generation when the amount of cocatalyst is increased up to a certain amount (in this case 5 nm), while a larger amount of cocatalyst (Pt film thickness ≥ 7–15 nm) leads to a significantly lower photocatalytic activity (Fig. 13c). It is evident that the trend of photocatalytic results is ascribed to an optimum of critical factors such as the density of induced Pt/TiO_2_ Schottky junctions, the free TiO_2_ surface *vs.* the TiO_2_ area coated with Pt NPs, and the light harvesting *vs.* shadowing effect.

## 6. Summary and outlook

Electrochemically-grown, highly periodic oxide structures, particularly self-organized anodic TiO_2_ nanotube arrays, are ideal surfaces for template-guided solid-state dewetting of thin metal films. By this approach, defined metal/oxide assemblies can be formed with nanoscale precision that have advanced functionalities, namely for photocatalysis and green hydrogen generation, ascribed not only to the inherent physico-chemical features of TiO_2_ but also to intimate metal–oxide interaction (metal–semiconductor coupling) and to their synergistically-achieved “double” self-ordering nature.

In this perspective we discussed the possibilities, limitations and solutions of using an ensemble of multilevel self-ordering principles to reach hierarchical nanoscopic designs. The approach is not only low cost but also scalable, and with high throughput, being completely based on self-ordering processes.

In the frame of photocatalytic applications, owing to the high cost of noble metal cocatalyst, a key challenge is to limit the use of Au and Pt cocatalysts. Dewetting work nicely demonstrates that the ideal conditions for an efficient photocatalytic process are not established by the use of a specific amount of noble metal, but rather by how effective is the cocatalyst/semiconductor junction design in order to satisfy a set of critical factors, such as the density of M/oxide junctions, free- *vs.* shaded-TiO_2_ surface, light harvesting *vs.* shadowing effects, and the M/ and TiO_2_/environment interface.

In other words, more than the cocatalyst amount as such, critical factors that need to be optimized for reasonable photocatalytic efficiency are the cocatalyst geometry and metal/TiO_2_ design, the control of which is only poor when using common photocatalyst syntheses and cocatalyst deposition methods.

There is still an enormous potential regarding the tailoring of TiO_2_ nanotube geometry, structure, wettability and doping, and the metal dewetting process will even provide a higher degree of designed functionalities. For example, additional self-ordering processes (spinodal decomposition, site-selective functionalization) and post-treatments (annealing in reactive atmosphere) can be interlaced to form more complex hierarchical assemblies (such as core–shell and nano-sponge structures, oxide–metal-molecular complexes).
